# Carbon fiber couches and skin sparing

**DOI:** 10.1120/jacmp.v11i2.3241

**Published:** 2010-04-16

**Authors:** Laurence Court, Jaime Urribarri, Mike Makrigiorgos

**Affiliations:** ^1^ Department of Radiation Oncology, Dana‐Faber / Brigham & Women's Cancer Center Harvard Medical School Boston MA 02115 USA

Dear Editor,

Over the last few years, medical LINAC manufacturers have started to offer couch tops or inserts designed for image‐guided radiation therapy (IGRT). These couch tops have a homogeneous construction, with a carbon fiber shell and no metal components. Although the use of these couches may offer some advantages in image quality, it can have a potentially serious impact on skin sparing.^(1)^ The characteristics of new carbon fiber couches have been reported in this journal previously,^(2)^ ^,^ ^(3)^ but the reports focus on couch transmission. We think the readers of the *Journal of Applied Clinical Medical Physics* may find our measurements of skin sparing for some modern couches useful when deciding on new couches. This data will supplement that of Higgins et al. who reported on a Sinmed BV couch.^(1)^

**Figure 1:**
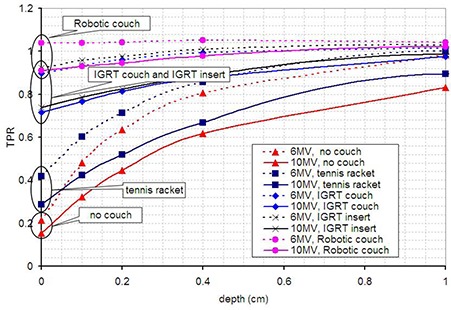
TPR in the build‐up region for 6 MV and 10 MV beams when using different carbon fiber couches.

We used a thin‐window parallel plate ion chamber (Capintec PS‐033) in solid water to measure the TPR in the build‐up region for 6 MV and 10 MV beams (10 by 10 cm field) with three different IGRT couches [IGRT insert for Exact couch (Varian), IGRT couch (Varian), Robotic Couch (BrainLAB)], with the regular tennis‐racket insert (Varian), and also with no couch in the beam. The results are shown in Fig. 1. All data is normalized at 10 cm depth to 0.775cGy/MU and 0.842cGy/MU for 6 MV and 10 MV beams, respectively. It can be seen in Fig. 1 that the use of carbon fiber couches reduces skin sparing significantly, and in one case eliminates it completely.

Although the loss of skin sparing is less important for multifield treatments, it might become an issue for AP‐PA or PA treatments that can comprise a significant fraction of treatments. For example, the use of a carbon fiber couch may increase the skin dose for a PA spine treatment from around 40% of prescription dose to 90–100% of the prescription dose, depending on the couch used. The skin dose for AP‐PA treatments may also increase to more than 90% of the prescription dose. Depending on the distribution of treatment sites and techniques and number of LINACs in their centers, physicists should consider including requirements on skin sparing in their couch specifications.
